# Supplementary figures and images for: CoRegNet: unraveling gene co-regulation networks from public RNA-Seq repositories using a beta-binomial statistical model
Source: Brief Bioinform. 2023 Dec 18;25(1):bbad380. doi: 10.1093/bib/bbad380 (PMC10729864; doi:10.1093/bib/bbad380)

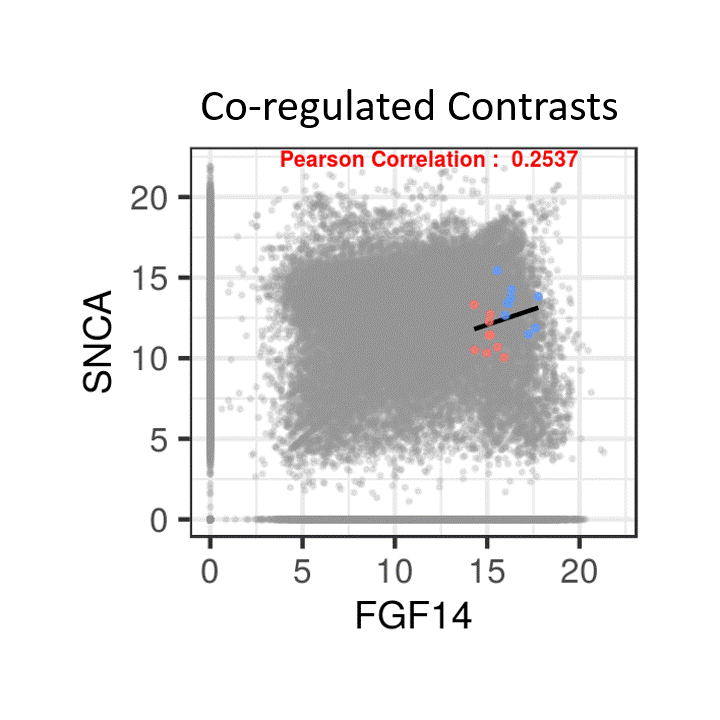

Supplement: Supplementary_animation_1_bbad380 [file supplementary_animation_1_bbad380.gif]
